# Effective population sizes of a major vector of human diseases, *Aedes aegypti*

**DOI:** 10.1111/eva.12508

**Published:** 2017-09-03

**Authors:** Norah P. Saarman, Andrea Gloria‐Soria, Eric C. Anderson, Benjamin R. Evans, Evlyn Pless, Luciano V. Cosme, Cassandra Gonzalez‐Acosta, Basile Kamgang, Dawn M. Wesson, Jeffrey R. Powell

**Affiliations:** ^1^ Yale University New Haven CT USA; ^2^ Fisheries Ecology Division Southwest Fisheries Science Center National Marine Fisheries Service and University of California Santa Cruz CA USA; ^3^ Centro Nacional de Programas Preventivos y Control de Enfermedades Ciudad de México CDMX Mexico; ^4^ LSTM/OCEAC Research Unit Organisation de Coordination pour la lutte contre les Endémies en Afrique Centrale Yaoundé Cameroon; ^5^ Department of Tropical Medicine Tulane University New Orleans LA USA

**Keywords:** arbovirus, chikungunya, dengue, effective population size, genetic control, temporal sampling, yellow fever, Zika

## Abstract

The effective population size (*N*_*e*_) is a fundamental parameter in population genetics that determines the relative strength of selection and random genetic drift, the effect of migration, levels of inbreeding, and linkage disequilibrium. In many cases where it has been estimated in animals, *N*_*e*_ is on the order of 10%–20% of the census size. In this study, we use 12 microsatellite markers and 14,888 single nucleotide polymorphisms (SNPs) to empirically estimate *N*_*e*_ in *Aedes aegypti*, the major vector of yellow fever, dengue, chikungunya, and Zika viruses. We used the method of temporal sampling to estimate *N*_*e*_ on a global dataset made up of 46 samples of *Ae. aegypti* that included multiple time points from 17 widely distributed geographic localities. Our *N*_*e*_ estimates for *Ae. aegypti* fell within a broad range (~25–3,000) and averaged between 400 and 600 across all localities and time points sampled. Adult census size (N_c_) estimates for this species range between one and five thousand, so the *N*_*e*_/*N*_*c*_ ratio is about the same as for most animals. These *N*_*e*_ values are lower than estimates available for other insects and have important implications for the design of genetic control strategies to reduce the impact of this species of mosquito on human health.

## 1 INTRODUCTION

The effective population size (*N*_*e*_) is a conceptual, idealized parameter, almost always much smaller that census size due to a number of demographic factors such as unequal sex ratios, population fluctuations, and unequal contribution to reproduction. *N*_*e*_ is a fundamental parameter in population genetics because the relative strength of selection and random genetic drift in populations as well as other basic properties such as the effect of migration and levels of genetic variation, inbreeding, and linkage disequilibrium scale with changes in *N*_*e*_. In many cases where it has been estimated, *N*_*e*_ is on the order of 10%–20% of the census size (Luikart, Ryman, Tallmon, Schwartz, & Allendorf, 2010; Palstra & Fraser, 2012).

The recent boom in the use of genetic methods to control transmission of vector‐borne diseases requires knowing the *N*_*e*_ of target vectors in order to design the interventions and predict their probability of success. *Aedes aegypti,* the major vector of yellow fever, dengue, chikungunya, and Zika viruses, has become a model for efforts of genetic control of disease vectors. Control programs may involve suppressing or genetically modifying populations to decrease their efficiency at transmitting pathogens (McGraw & O'Neill, 2013).

Accounting for *N*_*e*_ can improve vector control because *N*_*e*_ is not only directly related to census size and population structure, but is also a key parameter in modeling the rate of evolutionary change. A large *N*_*e*_ generally provides a buffer from the negative effects of inbreeding and allows for more rapid adaptive change by natural selection (Ohta, 1992; Olson‐Manning, Wagner, & Mitchell‐Olds, 2012). On the other hand, a small *N*_*e*_ increases the negative effects of inbreeding and the rate of fixation or loss of genetic variation by the process of genetic drift (Ohta, 1992). Strong genetic drift could cause even selectively advantageous alleles to drift out of populations over time. Thus, accounting for this key population parameter in *Ae. aegypti* control efforts can minimize risk of evolved resistance and maximize the spread and maintenance of traits that are desirable for reducing impact of *Ae. aegypti* on human health. Previous estimates from Australia, Thailand, and Indonesia using microsatellites, and estimates from Thailand using SNPs, have indicated *N*_*e*_ ranges from 11 to 5,564, suggesting relatively small breeding units regardless of the level of urban development (Endersby et al., 2011; Olanratmanee et al., 2013; Rašić et al., 2015).

During our ongoing worldwide survey of genetic variation in *Ae. aegypti* (Brown et al., 2011; Evans et al., 2015; Gloria‐Soria, Brown, Kramer, Yoshimizu, & Powell, 2014; Gloria‐Soria, Ayala, et al., 2016)*,* we have obtained temporal genetic data (microsatellites and SNPs) on samples from the same population separated by one to 7 years. Elsewhere, we reported on the genetic stability of populations over time relative to geographic differentiation (Gloria‐Soria, Kellner, et al., 2016). Here, we use these data to estimate *N*_*e*_ in 17 *Ae. aegypti* populations occupying a wide range of ecological settings from around the world. *N*_*e*_ can be estimated in various ways (Anderson, 2005; Jorde & Ryman, 2007; Krimbas & Tsakas, 1971; Luikart et al., 2010), and we have used several appropriate to our data (microsatellite allele frequencies and SNPs), life history (overlapping generations), and age of populations (young).

## 2 MATERIALS AND METHODS

### 2.1 Mosquito collections and DNA extraction

**Figure 1:**
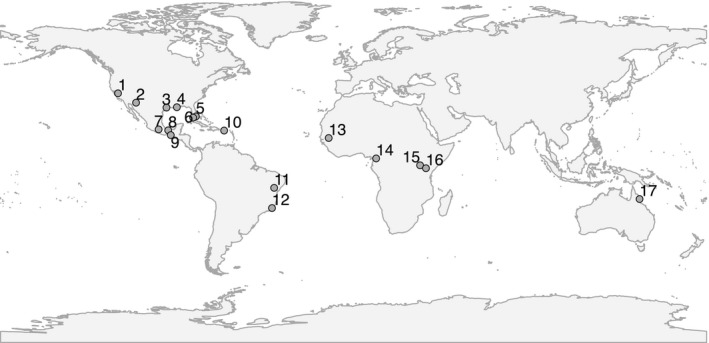
Sampled localities: (1) Madera, USA; (2) Tucson, USA; (3) Houston, USA; (4) New Orleans, USA; (5) Vaca Keys, USA; (6) Key West, USA; (7) Amacuzac, Mexico; (8) Coatzacoalcos, Mexico; (9) Pijijiapan, Mexico; (10) Patillas, Puerto Rico; (11) Jacobina, Brazil; (12) Cachoeiro, Brazil; (13) Goudiry, Senegal; (14) Yaounde, Cameroon; (15) Lunyo, Uganda; (16) Rabai, Kenya; and (17) Cairns, Australia

*Aedes aegypti* adults, larvae, or eggs were received from 17 localities worldwide (Table 1, Figure 1). When necessary, we completed additional laboratory work and scored microsatellite alleles and SNP‐chip genotypes following the same standards as for previous work reported from our lab. New samples arrived as either eggs from oviposition traps or as larvae/adults in 70%–100% ethanol from multiple traps or larval breeding sites to avoid sampling siblings. Eggs were hatched at the Yale School of Epidemiology and Public Health insectary, reared to adults, and stored in 100% ethanol at −20°C until DNA extraction. Genomic DNA was extracted using the DNeasy Blood and Tissue kit (Qiagen, Hilden, Germany), with a preliminary homogenization step in a TissueLyser II bead beater (Qiagen) and RNAse A (Qiagen).

**Table 1:** Two‐sample *N*_*e*_ estimates based on 12 microsatellites; locality, sampled years and sampled generations in parentheses counting from zero at the first time point sampled, harmonic mean number of mosquitos sampled (N), time interval spanning the two samples in generations (I), *N*_*e*_ estimates made with the Jorde and Ryman (2007) method in *NeEstimator v2* (Do et al., 2014) (*N*_*e*_^1^) with lower and upper 95% confidence intervals (CI^1^), and *N*_*e*_ estimates made with the Anderson (2005) method in *CoNe* (Anderson, 2005) (*N*_*e*_^2^) with lower and upper 95% confidence intervals (CI^2^)

Locality	Sampled years (generations)	*N*	I	*N*_*e*_^1^	Lower CI^1^	Upper CI^1^	*N*_*e*_^2^	Lower CI^2^	Upper CI^2^
01 Madera, USA	2013 & 2015 (0 & 12)	51.4	12	287.9	173.5	430.9	551.0	223.9	4860.4
02 Tucson, USA	2012 & 2013 (0 & 7)	53.5	7	48.5	30.8	70.3	90.2	56.4	149.8
	2012 & 2015 (0 & 21)	54.0	21	131.8	85.7	187.6	392.4	243.8	661.2
	2013 & 2015 (7 & 21)	53.5	14	1172.8	759.0	1675.2	∞	∞	∞
03 Houston, USA^a^	2009 & 2011 (0 & 18)	23.0	18	25.0	14.4	45.3	37.8	26.3	55.0
04 New Orleans, USA	2011 & 2012 (0 & 9)	53.2	9	84.0	57.9	115.0	604.6	289.6	2627.5
	2011 & 2014 (0 & 27)	50.5	27	493.2	332.8	684.8	2233.4	916.3	∞
	2011 & 2015 (0 & 36)	37.7	36	223.2	148.9	312.2	548.9	327.2	1020.7
	2012 & 2014 (9 & 27)	59.3	18	441.1	305.5	601.2	938.4	487.9	2661.2
	2012 & 2015 (9 & 36)	42.4	27	269.9	187.5	367.2	401.7	242.9	727.7
	2014 & 2015 (27 & 36)	40.7	9	162.6	111.2	223.5	197.7	108.4	454.7
05 Vaca Keys, USA	2006 & 2009 (0 & 36)	42.5	36	233.1	152.4	330.9	458.5	287.3	776.3
	2006 & 2015 (0 & 84)	45.4	84	1180.5	775.1	1670.1	1796.0	1048.0	3605.4
	2009 & 2015 (36 & 84)	44.8	48	253.8	167.4	358.0	570.9	378.7	896.5
06 Key West, USA	2009 & 2011 (0 & 24)	30.0	24	187.6	125.7	261.8	315.6	185.7	621.9
	2009 & 2013 (0 & 48)	38.8	48	404.2	274.8	558.4	775.7	481.8	1398.4
	2009 & 2016 (0 & 84)	38.8	84	2662.0	1783.6	3714.0	2888.6	1382.0	10506
	2011 & 2013 (24 & 48)	37.2	24	84.6	56.2	118.6	242.9	163.4	374.2
	2011 & 2016 (24 & 84)	37.2	60	314.3	208.2	442.1	750.7	482.5	1242.5
	2013 & 2016 (48 & 84)	52.0	36	500.6	331.5	704.1	752.2	457.6	1366.2
07 Amacuzac, MX	2012 & 2013 (0 & 16)	54.0	16	184.5	113.5	272.5	222.0	132.0	400.4
	2012 & 2014 (0 & 24)	53.5	24	260.4	162.4	381.3	250.7	154.8	417.7
	2012 & 2016 (0 & 48)	53.0	48	310.5	191.0	458.5	487.2	295.9	831.3
	2013 & 2014 (16 & 24)	53.5	8	43.4	26.3	64.6	67.7	43.9	106.3
	2013 & 2016 (16 & 48)	53.0	32	174.9	105.4	261.8	258.7	165.7	412.9
	2014 & 2016 (24 & 48)	52.5	24	98.0	59.1	146.7	177.6	114.3	281.8
08 Coatzacoalcos, MX^a^	2003 & 2008 (0 & 60)	41.2	60	47.3	27.9	71.9	65.7	46.8	91.4
09 Pijijiapan, MX	2006 & 2008 (0 & 24)	47.5	24	82.0	44.8	130.2	161.0	100.9	257.2
10 Patillas, PR	2012 & 2014 (0 & 24)	54.0	24	159.3	102.1	229.0	180.3	121.1	272.4
11 Jacobina, BR	2013 & 2014 (0 & 8)	60.5	8	38.8	27.4	52.2	91.3	60.2	141.9
	2013 & 2015 (0 & 14)	59.5	14	114.5	72.7	165.7	281.8	173.2	507.1
	2014 & 2015 (8 & 14)	60.0	6	226.2	147.8	321.0	58.0	39.8	85.5
12 Cachoeiro, BR^a^	2008 & 2010 (0 & 24)	30.9	24	40.0	25.3	58.1	174.8	118.7	267.9
	2008 & 2012 (0 & 48)	30.9	48	240.2	150.7	350.4	696.5	412.1	1403.7
	2010 & 2012 (24 & 48)	47.0	24	47.1	30.3	67.5	106.3	76.6	148.6
13 Goudiry, SE^a^	2007 & 2012 (0 & 60)	49.7	60	82.4	53.3	117.7	150.4	117.5	191.6
14 Yaounde, CM	2009 & 2014 (0 & 55)	50.3	55	232.4	168.2	306.9	520.9	394.1	691.7
	2009 & 2015 (0 & 69)	50.7	69	485.9	352.4	640.6	1023.0	739.2	1453.3
	2014 & 2015 (55 & 69)	54.5	14	72.3	52.3	95.4	178.4	133.1	244.6
15 Lunyo, UG^a^	2012 & 2013 (0 & 12)	53.5	12	35.3	24.9	47.6	71.2	54.3	93.5
16 Rabai, KE	2006 & 2009 (0 & 36)	33.7	36	724.9	543.0	932.7	3549.5	1317.9	∞
	2006 & 2012 (0 & 72)	21.1	72	202.9	148.3	266.0	228.8	161.1	331.6
	2009 & 2012 (36 & 72)	22.3	36	109.6	80.4	143.3	121.5	85.0	177.6
17 Cairns, AU	2009 & 2013 (0 & 48)	49.5	48	292.6	185.7	423.6	618.6	396.4	1006.7
	2009 & 2015 (0 & 62)	46.5	62	328.2	203.3	482.6	552.0	358.3	878.5
	2009 & 2015 (0 & 62)	47.8	14	305.4	189.2	449.0	193.2	111.3	365.8

a Locality with evidence of temporal shifts determined by principal components analysis (Fig. S1) and neighbor‐joining phylogenetic analysis (Fig. S2).

### 2.2 Genotyping 12 microsatellites and 14,888 SNPs

For the microsatellite genotyping, we used the protocol described in (Brown et al., 2011) for 12 loci; A1, B2, B3, A9, AC2, CT2, AG2, AC4, AC1, AC5, AG1, and AG4. Briefly, amplifications were performed using standard PCR protocol (35 cycles at 54°C) with fluorescently labeled M13 primers (6‐FAM and HEX) in 10.0 μl reaction volumes using the Type‐it Microsatellite PCR Master Mix (Qiagen), then diluted, multiplexed, and submitted for fragment analysis with GS 500 ROX internal size standard (Applied Biosystems, Foster City, CA, USA) on an Applied Biosystems 3730xl DNA Genetic Analyzer at the DNA Analysis Facility on Science Hill at Yale. Alleles were scored using GeneMapper v4.0 (Applied Biosystems).

For the SNP‐chip genotyping, 167 samples were analyzed on the Axiom_aegypti1 SNP‐chip (Evans et al., 2015) at the Functional Genomics Core at University of North Carolina, Chapel Hill, using manufacturer protocols. Raw data were processed and converted into genotype calls following Evans et al. (2015) using *Genotyping Console v4.2* (Affymetrix, Santa Clara, CA, USA) and *SNPolisher v1.4* (Affymetrix) in the R environment with the call threshold set to 95%. SNPs were pruned to remove any linked SNPs in *PLINK v1.7* (Purcell et al., 2007) with the command ‘–indep 100 10 2′, which recursively removed SNPs within a sliding window of 100 SNPs wide, shifting 10 SNPs per step, with a variance inflation factor (i.e., VIF) threshold of 2.

### 2.3 Assessment of temporal stability and sibling relationships

Previous work has shown that some *Ae. aegypti* populations have undergone temporal shifts in allele frequencies (Gloria‐Soria, Kellner, et al., 2016). To identify any regions in this larger dataset where whole populations might have received an influx of migrants or otherwise been disrupted in a way that would make temporal methods of *N*_*e*_ estimation difficult to apply (Luikart et al., 2010), we estimated population structure among and between multiple time points using principal components analysis (PCA), and neighbor‐joining phylogenetic analysis. We subjected all microsatellite data to principal components analysis (PCA) with the ‘adegenet’ package v 1.4‐2 (Jombart, 2008) in the *R v3.0.2* environment (R Development Core Team, 2013), and visualized using *JMP v11.0* (SAS Institute Inc., Cary, NC, USA). We then estimated the optimal neighbor‐joining (NJ) tree (Saitou & Nei, 1987) of genetic distances (Cavalli‐Sforza & Edwards, 1967) with support values based on 1000 bootstrap replicates using *NEIGHBOR* implemented in *PHYLIP v3.69* (Felsenstein, 1989, 2005).

To identify instances where the presence of related individuals could artificially increase the variance in the estimated allele frequencies and thus decrease the estimates of *N*_*e*_, we identified full siblings using COLONY v2.0.6.3 (Jones & Wang, 2010). We then performed estimates of *N*_*e*_ with siblings removed for comparison with our main results. For this dataset, we randomly removed all but two individuals for each inferred full sibling group.

### 2.4 Estimates of *N*_*e*_

*N*_*e*_ was estimated with the two‐sample temporal methods (Waples, 1989) based on coalescence theory in *CoNe* (Anderson, 2005) and based on F‐statistic moments (Jorde & Ryman, 2007) in *NeEstimator v2* (Do et al., 2014), as well as with the single‐sample LD method (Waples & Do, 2008), also in *NeEstimator v2*. These estimates complement one another because they represent the three main types of *N*_*e*_ estimators (coalescence *N*_*e*_, variance *N*_*e*_, and inbreeding *N*_*e*_, respectively), thus having different strengths, weaknesses, and known biases. For example, the two‐sample coalescence method of Anderson (2005) and the F‐statistic moments method of Jorde and Ryman (2007) are robust in the case of overlapping generations and can deal with lower levels of polymorphisms (Luikart et al., 2010), but they calculate a single estimate across two time points and so are vulnerable to gene flow and fixation of rare alleles during the time interval between samples (Jorde & Ryman, 2007; Anderson, 2005), whereas the LD method (Waples & Do, 2008) is less vulnerable to gene flow and fixation of rare alleles, but runs the risk of bias caused by overlapping generations. Moreover, it does not provide enough power to distinguish from infinite population sizes in the case of insufficient polymorphisms (Hill, 1981; Waples & Do, 2008).

We estimated 95% confidence intervals using the points where the log‐likelihood dropped 1.96 units from the maximum in *CoNe* (Anderson, 2005), and using the parametric method in *NeEstimator v2* (Do et al., 2014). The number of generations per year used (Table S1) equaled the number of months of the year wherein monthly average minimum temperature was above 10°C in 2013 according to Weather Underground's (The Weather Company, San Francisco, CA, USA) closest station. This estimate was based on experimental evidence that *Ae. aegypti* eggs do not develop at temperatures below 10°C (Christophers, 1960).

## 3 RESULTS

**Figure 2:**
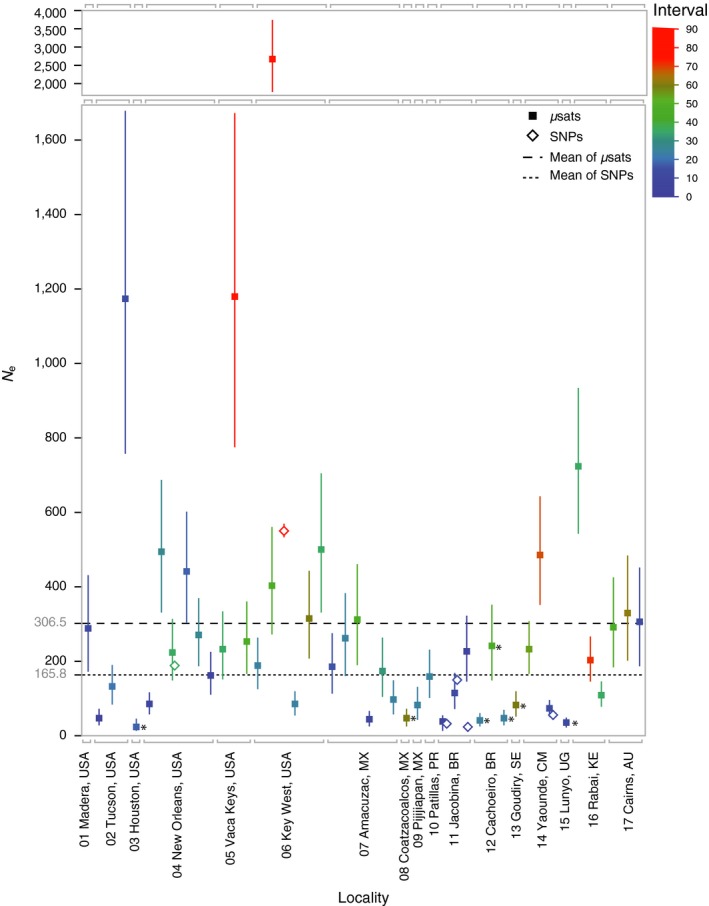
Two‐sample *N*_*e*_ estimates made with the Jorde and Ryman (2007) method in *NeEstimator v2* (Do et al., 2014) and with the Anderson (2005) method in *CoNe* (Anderson, 2005). Mean effective population size estimates (*N*_*e*_), lower and parametric 95% confidence interval (CI) are displayed by locality, colored by the number of generations spanning the two samples used in each estimate (generations spanned). The average *N*_*e*_ across all estimates of each data type (μsats in dashed and SNPs in dotted) is displayed as a horizontal line. Estimates from localities with evidence of temporal shifts determined by principal components analysis (Fig. S1) and neighbor‐joining phylogenetic analysis (Fig. S2) are marked with an asterix (*)

Estimates of effective population size (*N*_*e*_) of *Aedes aegypti* from 17 localities (Figure 1) and 47 time points (Table S1) obtained through multiple methods indicate small breeding units that ranged from 25 to 3610 and averaged less than 600 individuals (Table 1). The Jorde and Ryman (2007) method yielded estimates that averaged 290.3 (Table 1, Figure 2), while the Anderson (2005) method yielded generally higher estimates (Fig. S3) that averaged 535.1 (Table 1). These results are in line with previous studies in *Ae. aegypti* conducted at local geographic scales (Endersby et al., 2011; Olanratmanee et al., 2013; Rašić et al., 2015) and suggest localized breeding units even where regional census size is large.

### 3.1 Assessment of temporal stability and sibling relationships

We found evidence of temporal disruptions in Houston, Coatzacoalcos, Cachoeiro, Goudiry, and Lunyo. Evidence included separation of multiple time points along the first four axes of the PCA (Fig. S1), and closer relationships between distant geographic locations than between multiple time points from the same location in neighbor‐joining phylogenetic analysis (Fig. S2). We compared results with exclusion of these localities that have a heightened risk of violation of the assumptions to confirm consistency of results. Results from COLONY indicate the presence of siblings in the samples at some localities such as Patillas and Lunyo (Table S1), but very few siblings in many localities. We compare results with exclusion of siblings to confirm that conclusions of the study were not impacted and find there is no significant difference (*t*‐test *p*‐value .4462) in mean estimates with and without sibling removal (Fig. S5).

### 3.2 Estimates of *N*_*e*_ based on microsatellite data

We estimated *N*_*e*_ with two different temporal methods that are robust to the potential bias introduced by overlapping generations (Luikart et al., 2010; Waples, 1989). We combined datasets previously generated in our laboratory at Yale University (Brown et al., 2011; Gloria‐Soria et al., 2014; Gloria‐Soria, Ayala, et al., 2016; Gloria‐Soria, Kellner, et al., 2016; Monteiro et al., 2014; Pless et al., 2017 in review) with newly genotyped mosquitos. The final microsatellite dataset included 12 loci from an average of 46.7 individuals per time point sampled (Table S1). *N*_*e*_ estimates from the Jorde and Ryman (2007) method implemented in *NeEstimator v2* (Do et al., 2014) averaged 303.3 (Figure 2) and ranged from 25.0 to 1181.0 with the exception of a single outlier of 2662.0 (*N*_*e*_^1^ in Table 1), with narrow 95% confidence intervals that ranged from an absolute low of 14.4 (lower CI^1^ in Table 1) to an absolute high of 3714.0 (upper CI^1^ in Table 1). *N*_*e*_ estimates with the Anderson (2005) method were not significantly different, but were on average 1.32 times higher (Fig. S3), and averaged 515.4 and ranged from 37.9 to indistinguishable from infinite (*N*_*e*_^2^ in Table 1), with 95% confidence intervals that spanned from an absolute low of 26.3 (lower CI^2^ in Table 1) to a high of infinite (upper CI^2^ in Table 1). As expected, localities with evidence of temporal disruptions had smaller *N*_*e*_ estimates than genetically stable localities, but removal of these few localities increased average *N*_*e*_ estimates only slightly to 349.8 and 626.5 (Table 1).

To determine whether variation in the length of the time interval between collections and the number of generations per year used in the calculations introduced bias, we conducted a t‐test that confirmed that number of generations per year did not significantly impact *N*_*e*_ estimates for either temporal method (*p*‐value of .8892 and .2556). However, the length of the time interval between samples was significantly correlated with *N*_*e*_ (Fig. S2) with an R^2^ value of 0.23 for the Jorde and Ryman (2007) method (*p*‐value .0007; Fig. S2A), and with an R^2^ value of 0.18 with the Anderson (2005) method (*p*‐value .0033; Fig. S2B). Removing localities with evidence of temporal disruption did not reduce significance of this correlation nor did removing outliers.

### 3.3 Estimates of *N*_*e*_ based on SNP data

Estimates of *N*_*e*_ based on single nucleotide polymorphisms (SNPs) were completed with the same two methods that we used for microsatellites; the Jorde and Ryman (2007) and Anderson (2005) methods. The dataset was a combination of newly genotyped samples and previously published data from Evans et al. (2015) and included 14,888 SNPs from an average of 15.9 individuals per time point. *N*_*e*_ estimates with the Jorde and Ryman (2007) method averaged 166.0 (Figure 2) and ranged from 22.9 to 549.2 (*N*_*e*_^1^ in Table 2) with extremely narrow 95% confidence intervals that ranged from an absolute low of 22.4 (lower CI^1^ in Table 2) to an absolute high of 563.3 (upper CI^1^ in Table 2). *N*_*e*_ estimates with the Anderson (2005) method were not significantly different, but were on average 2.26 times higher (Fig. S3), and averaged 375.2 and ranged from 33.6 to 977.1 (*N*_*e*_^2^ in Table 2) with 95% confidence intervals that spanned from an absolute low of 32.0 (lower CI^2^ in Table 2) to an absolute high of 1214.3 (upper CI^2^ in Table 2).

**Table 2:** Two‐sample *N*_*e*_ estimates based on 14,888 SNPs; locality, sampled years and sampled generations in parentheses counting from zero at the first time point sampled, harmonic mean number of mosquitos sampled (*N*), time interval spanning the two samples in generations (I), *N*_*e*_ estimates made with the Jorde and Ryman (2007) method in *NeEstimator v2* (Do et al., 2014) (*N*_*e*_^1^) with lower and upper 95% confidence intervals (CI^1^), and *N*_*e*_ estimates made with the Anderson (2005) method in *CoNe* (Anderson, 2005) (*N*_*e*_^2^) with lower and upper 95% confidence intervals (CI^2^)

Locality	Sampled years (generations)	*N*	I	*N*_*e*_^1^	Lower CI^1^	Upper CI^1^	*N*_*e*_^2^	Lower CI^2^	Upper CI^2^
04 New Orleans	2012 & 2015 (9 & 36)	11.0	27	186.9	182.1	191.7	267.2	247.5	292.5
06 Key West	2009 & 2016 (0 & 84)	12.0	84	549.2	535.2	563.3	620.2	592.0	645.0
11 Jacobina	2013 & 2014 (0 & 8)	21.3	8	33.6	35.5	33.6	33.6	32.0	35.0
	2013 & 2015 (0 & 14)	20.3	14	147.5	144.0	151.1	977.1	700.0	1214.3
	2014 & 2015 (8 & 14)	14.5	6	22.9	22.4	23.5	138.4	120.0	145.0
14 Yaounde	2014 & 2015 (55 & 69)	15.5	14	54.8	53.5	56.1	214.6	198.3	233.3

### 3.4 Estimates of *N*_*e*_ based on single samples

To confirm that estimates using the two‐sample temporal methods used were not low‐biased because of undetected temporal disruptions between sampling points, we also used a single‐sample method based on linkage disequilibrium (LD) developed by Waples and Do (2008) in *NeEstimator v2* (Do et al., 2014). These *N*_*e*_ estimates ranged from 1.4 to 2526.3 with the exception of a single estimate indistinguishable from infinite, had a mean of 116.7 and a large variance with 95% confidence intervals that overlapped with infinity in about 15% of the estimates (Table S2). This indicates that single‐sample estimates are lower than the two‐sample estimates and strengthens the evidence that two‐sample temporal methods used were not low‐biased due to violation of assumptions.

## 4 DISCUSSION

Estimates of *N*_*e*_ of the *Aedes aegypti* mosquito ranged from ~25 to ~3,000 and averaged between 400 and 600 (Table 1, Figure 2). These results indicate relatively small breeding units for *Ae. aegypti* compared to most insects, including other mosquitoes. For example, both the census size and *N*_*e*_ of *Anopheles gambiae* (s.l.) in Africa have been estimated to be an order of magnitude greater than the estimates for *Ae. aegypti* presented here (Lehmann, Hawley, Grebert, & Collins, 1998; Taylor, Toure, Coluzzi, & Petrarca, 1993). This has immediate implications in design of successful genetic control programs. For example, it should be easier to genetically modify populations with smaller effective population sizes compared to larger ones, regardless of the type of modification use.

Estimating *N*_*e*_ in natural populations is difficult and subject to errors for a number of reasons. First, populations may experience considerable migration between sampling time points or even replacement. Our PCA and phylogenetic analysis (Fig. S1 and S2) indicated that *N*_*e*_ estimates in five of the seventeen localities may be impacted by such temporal disruptions. Indeed, these localities (Houston, Coatzacoalcos, Cachoeiro, Goudiry, and Lunyo) showed lower *N*_*e*_ estimates on average (Table 1, Figure 2). Low *N*_*e*_ in these localities may have been caused by violations of the assumption that allele frequency changes are due exclusively to genetic drift rather than migration or population subdivisions. Nonetheless, removal of localities with suspected temporal disruptions increased average *N*_*e*_ estimates only slightly (Table 1), indicating consistency of results. *N*_*e*_ estimates after removal of siblings showed that in some cases, the presence of siblings in the samples probably caused a small reduction in the inferred *N*_*e*_ (Table S4), as one would expect as the presence of related individuals will increase the variance in the estimated allele frequencies. However, in many cases there was almost no effect, and there was no significant difference in the overall mean of estimates (Table S4, Fig. S5).

Second, there was an indication in our data that there was an effect of length of time interval between sampling points on the *N*_*e*_ estimates; longer intervals produced larger *N*_*e*_ estimates (Fig. S4). This suggests bias in samples separated by time intervals between 10 and 84 generations. This bias is an expected outcome of the Jorde and Ryman (2007) algorithm due to the fixation of rare alleles during the interval sampled. However, an improvement of the original method (Jorde & Ryman, 1995) made in 2007 was meant to correct this bias (Jorde & Ryman, 2007). Our results suggest this correction did not completely remove the bias; however, they tested intervals up to only 10 generations, while our samples span up to an estimated 84 generations. On the other hand, the Anderson (2005) method should be less biased by fixation of alleles than a moment based estimator like the one of Jorde and Ryman (2007) because it does not rely on an approximate linear relationship between the magnitude of allele frequency change and genetic drift. Our results suggest that there may be some bias even with the Anderson (2005) method and indicate a need for further investigation of this issue.

Third, while we argue that the two‐sample temporal method is generally better than single‐sample estimates of *N*_*e*_, we did consider these latter methods, and the results were very similar. Although the single‐sample *N*_*e*_ estimates are somewhat lower (Table S2), this is added evidence that our two‐sample results indicating relatively small *N*_*e*_ are robust as they are comparable across completely independent estimation methods.

Although lower than estimates in most other insects, our results are consistent with estimates made in previous studies of this species of mosquito, *Ae. aegypti* (Table S3). Work in Northern Australia based on microsatellites found that *N*_*e*_ averaged 692 (Endersby et al., 2011). Work in Indonesia based on microsatellites and SNPs found that *N*_*e*_ averaged 467 excluding one infinite estimate (Rašić et al., 2015). Finally, work in Thailand based on microsatellites and EPIC found that *N*_*e*_ averaged 166 (Olanratmanee et al., 2013).

Estimates of census size (N_c_) for adult *Ae. aegypti* using mark–recapture methods range from about 900 for villages in Rabai, Kenya (Lounibos, 2003) to 5,500 for a city in Brazil (Carvalho et al., 2015). The most intensive mark–recapture study on *Ae. aegypti* was carried out by Sheppard, Macdonald, Tonn, and Grab (1969) who performed 23 releases over a full year in Bangkok, Thailand. The mean census size was 2,562 (both sexes) with a *SD* of 1,351 (Sheppard et al., 1969). *Ae. aegypti* census size has also been estimated by larval and pupal counts, but these are likely gross overestimates because they do not consider low survival rates to adulthood. For example, Dye (1984) found that less than 20% of larvae survive to mid‐pupal stage (Dye, 1984). Thus, we feel the studies cited above using adult mark–recapture methods are the best indicator of census size of adult breeders, the relevant comparison to *N*_*e*_. Our estimates, and previous ones, of *N*_*e*_ in the range of 100‐700 and of N_c_ from one to five thousand, means that *N*_*e*_ */N*_*c*_ for this species is 10%–30%—in line with most animals.

Interestingly, as pointed out above, the mosquito *An. gambiae* has been estimated to have an N_e_ about an order of magnitude greater than *Ae. aegypti*, N_c_ for *An. gambiae* has been estimated to be nearly an order of magnitude greater than these N_c_ estimates for *Ae. aegypti* (Touré et al., 1998). So despite the large difference in absolute population sizes, *N*_*e*_ */N*_*c*_ for these two mosquitoes remains very similar. This suggests that estimates of *N*_*e*_ can serve as reliable predictors of relative N_c_ and *vice versa,* factors relevant to planning and implementing genetic control programs.

The relatively small estimates of *N*_*e*_ reported here for *Ae. aegypti* almost certainly reflect the relatively short range of active dispersal of this mosquito (Harrington et al., 2005; Maciel‐De‐Freitas, Codeço, & Lourenco‐De‐Oliveira, 2007; Muir & Kay, 1998; Russell, Webb, Williams, & Ritchie, 2005), but see (Reiter, 2007). The results are consistent with a patchy metapopulation structure, sensu Harrison (1991), with localized breeding units even when quasi‐continuously distributed at a larger scale. For example, our samples from Yaounde, Cameroon, came from a single neighborhood and the estimated *N*_*e*_ (263 and 574 for the two methods) cannot represent the entire 180 km^2^ of available habitat in this city of 2.5 million people.

## 5 CONCLUSION

In summary, we have shown that *N*_*e*_ in *Ae. aegypti* is relatively small across our worldwide sample (Figure 2), suggesting that these mosquitos form localized breeding units even in large cities where the regional census size is large. This is important because *Ae. aegypti* has become a model system in design of control programs using genetic methods that aim to suppress or genetically modify populations to decrease their efficiency at transmitting pathogens (McGraw & O'Neill, 2013). Methods of genetically modifying vector populations that rely on inundation and replacement (e.g., that of Powell & Tabachnick, 2014) are quite feasible with such small populations. On the other hand, such small breeding units must be quite spatially limited. This means genetic modification over a larger area will require many local releases spatially separated across a target area. Even those genetic modifications based on gene drive would need to be seeded in many locations across a target area. The very slow spread of successful *Wolbachia* replacement in local sites in an Australian city is consistent with this view of *Ae. aegypti* population structure (Schmidt et al., 2017).

These estimates of *N*_*e*_ also indicate that genetic drift is quite strong in *Ae. aegypti* consistent with the remarkable population genetic differentiation observed for neutral markers (Brown et al., 2011; Gloria‐Soria, Ayala, et al., 2016; Powell & Tabachnick, 2014). This strength of drift needs to be considered in genetic modification programs. Even selectively advantageous alleles could drift out of populations over time in such small populations, suggesting a need for repeated releases and long‐term monitoring.

## DATA ARCHIVING STATEMENT

Microsatellite alleles and SNP‐chip genotypes used in our analyses have been deposited into the DRYAD database in ‘GenePop’ format (https://doi.org/10.5061/dryad.3v2v5)

## AUTHOR CONTRIBUTIONS

J.R.P. involved in the conceptualization; N.P.S., A.G., B.R.E., E.P., and J.R.P. involved in methodology; C.G.A., B.K., and D.M.W. involved in the field work; N.P.S., A.G., E.C.A, B.R.E., and E.P. investigated the study; N.P.S. and J.R.P. wrote the original draft of the manuscript; A.G., E.C.A, B.R.E., E.P., C.G.A, B.K., and D.M.W. involved in writing of the manuscript—review and editing; N.P.S. and E.C.A. involved in visualization; N.P.S., A.G., and J.R.P. involved in the project administration; J.R.P. involved in funding acquisition.
