# Supplementary material for: Effective population sizes of a major vector of human diseases, Aedes aegypti
Source: Evol Appl. 2017 Sep 3;10(10):1031–9. doi: 10.1111/eva.12508 (PMC5680635; doi:10.1111/eva.12508)
Supplement: Supplementary file 1 [file EVA-10-1031-s001.pdf]

Supplemental Figures and Legends

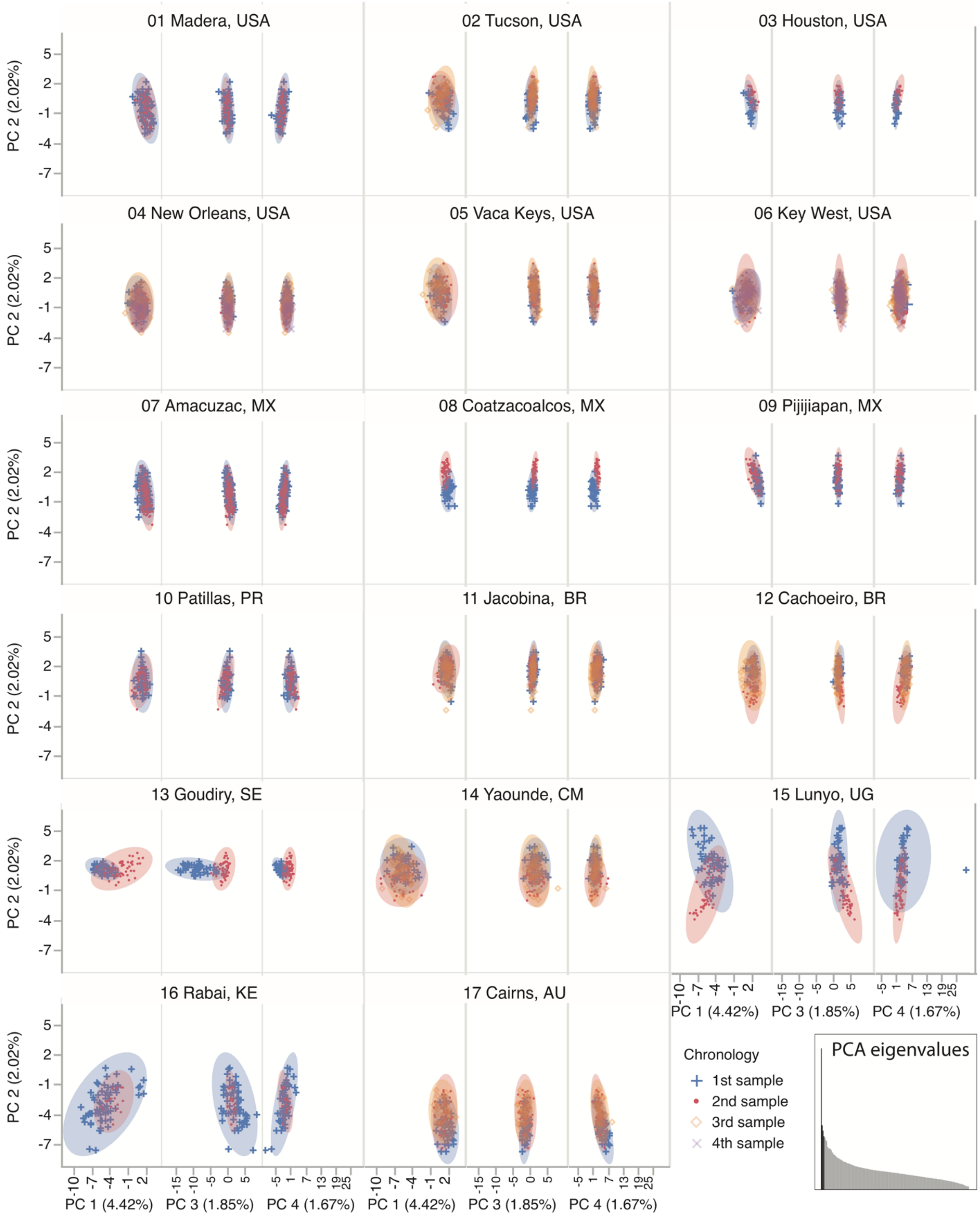

Figure S1. See also Table 1. Principle components analysis (PCA) showing principle component (PC) versus 1, 3, and 4 with ellipses covering 99% of each sample. Each year has its own marker and color in chronological order (blue "+", red dot, orange triangle, or purple “x”). PCA was conducted with the "adegenet" package v1.4-2 (Jombart, 2008) in the *R v3.0.2* environment (R Core Development Team, 2013) and visualized using *JMP v11.0* (SAS Institute Inc., Cary, NC, USA, 1989– 2007).

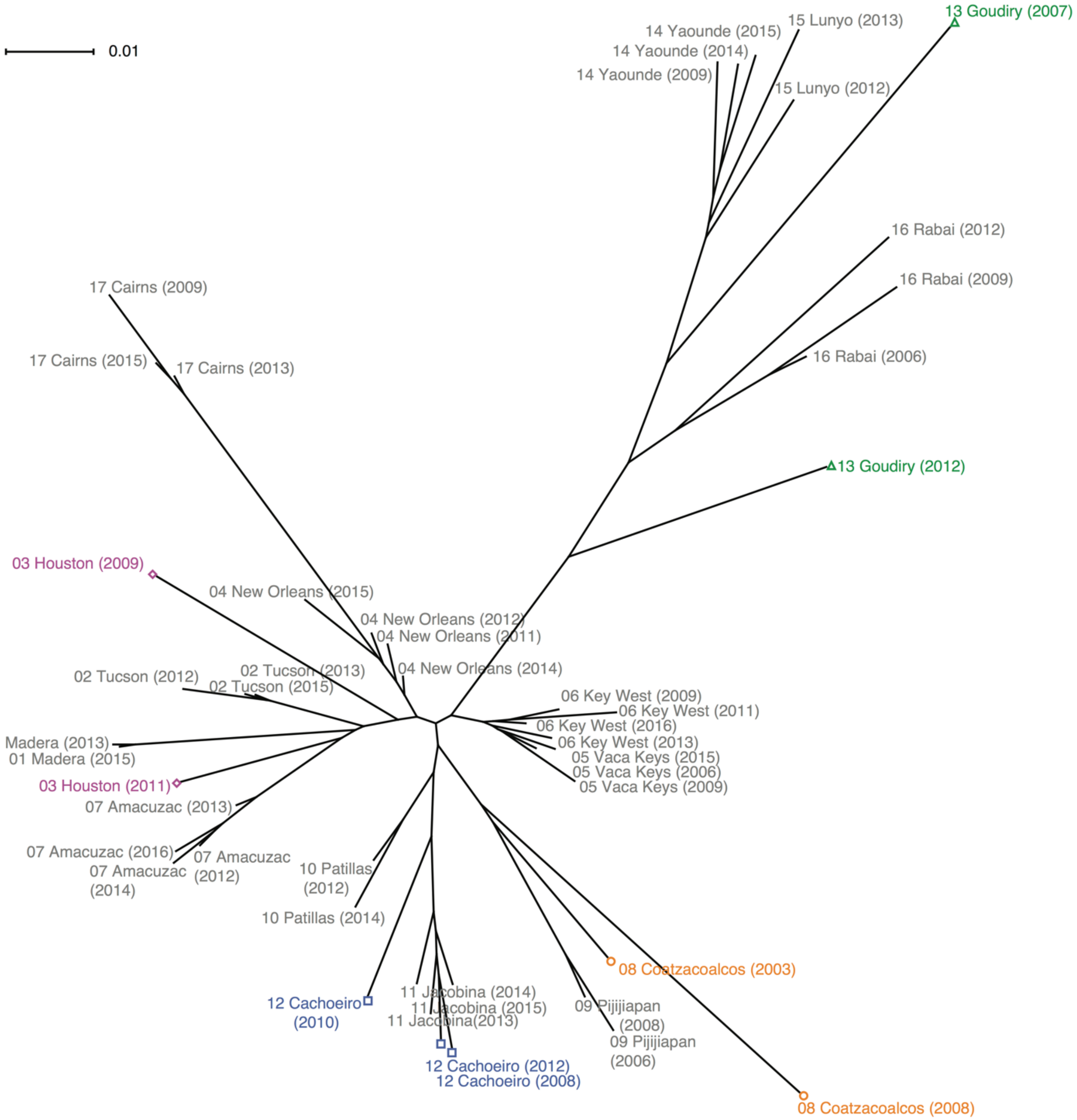

Figure S2. See also Table 1. Neighbor-joining tree with support values based on 1000 bootstraps using *NEIGHBOR* implemented in *PHYLIP v3.69* (Felsenstein, 1989, 2005). Candidate localities of temporal disruptions are marked with purple diamonds (Houston), orange circles (Coatzacoalcos), blue squares (Cachoeiro), or green diamonds (Goudiry).

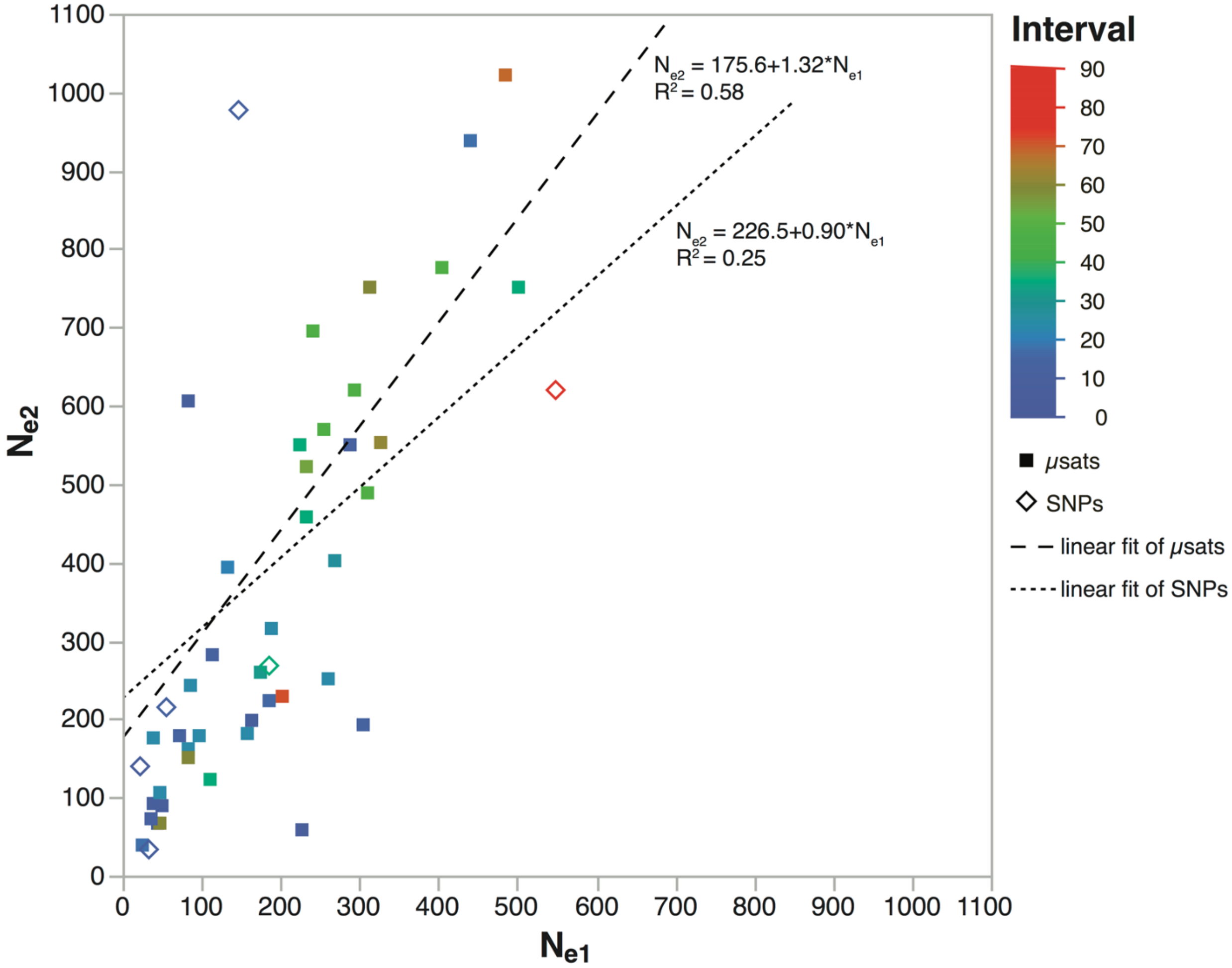

Figure S3. See also Figure 2. Comparison of N_e estimates from two temporal methods: The Jorde and Ryman (2007) method from *NeEstimator v2* (Do et al, 2014) shown on the X-axis (N_{e1}), and the Anderson (2005) from *CoNe* (Anderson, 2005) shown on the Y-axis (N_{e2}). Data from 12 microsatellites (µsats) shown with solid squares, and estimates from 14,888 single nucleotide polymorphisms (SNPs) shown with diamonds. The linear equation, the R^2 value, and the p-value of the linear fit of each data type (µsats in dashed and SNPs in dotted) was fit separately and is displayed within the plot.

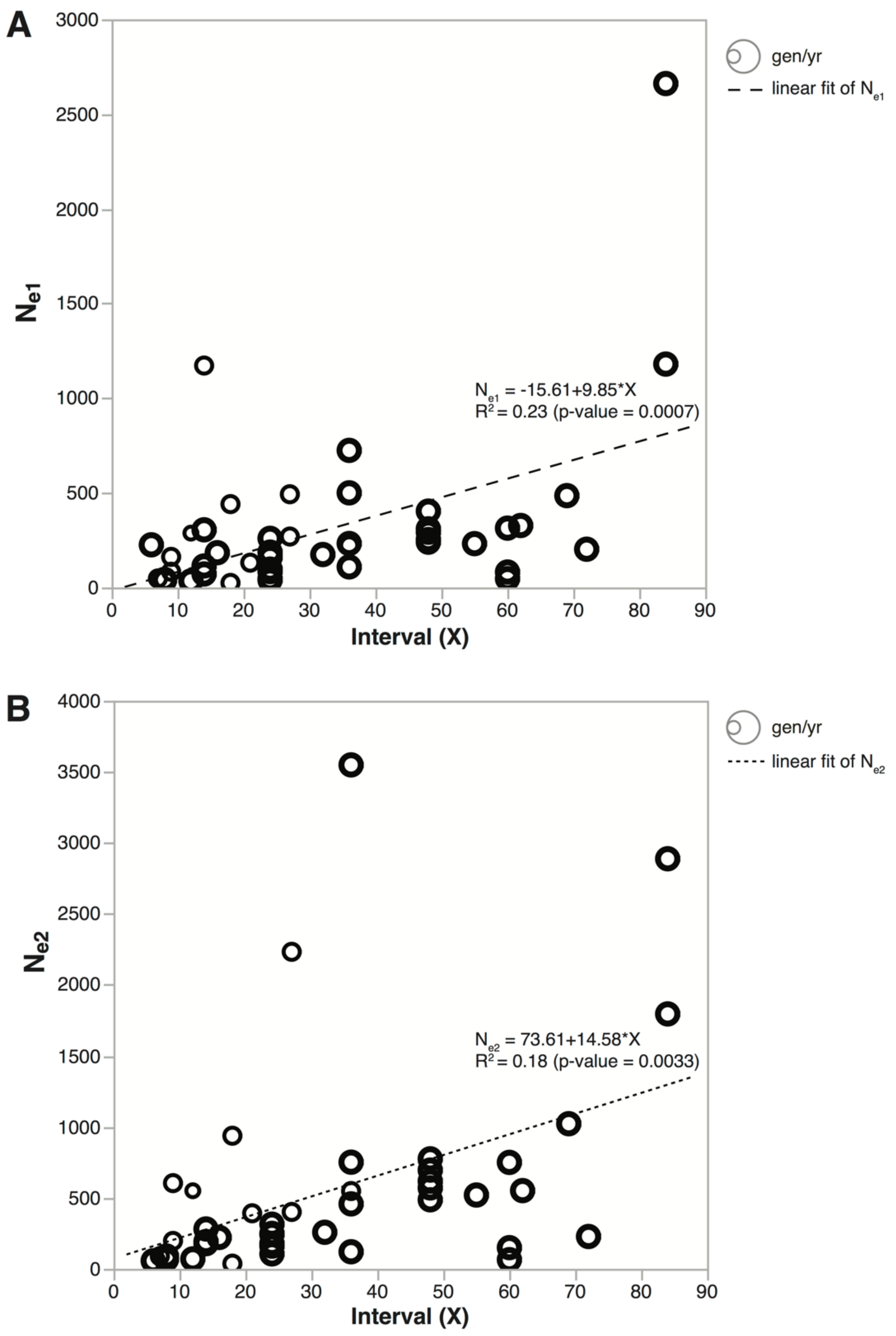

Figure S4. See also Figure 2. Linear regression N_e by time interval (in generations) between samples, sized by the number of generations per year (see Table 1 for details). N_e estimates from **(A)** the Jorde and Ryman (2007) method in *NeEstimator v2* (Do et al, 2014), and **(B)** the Anderson (2005) method in *CoNe* (Anderson, 2005). The linear equation, the R^2 value, and the p-value of the linear fit of each method (N_{e1} in dashed and N_{e2} in dotted) was fit separately and is displayed within the plot.

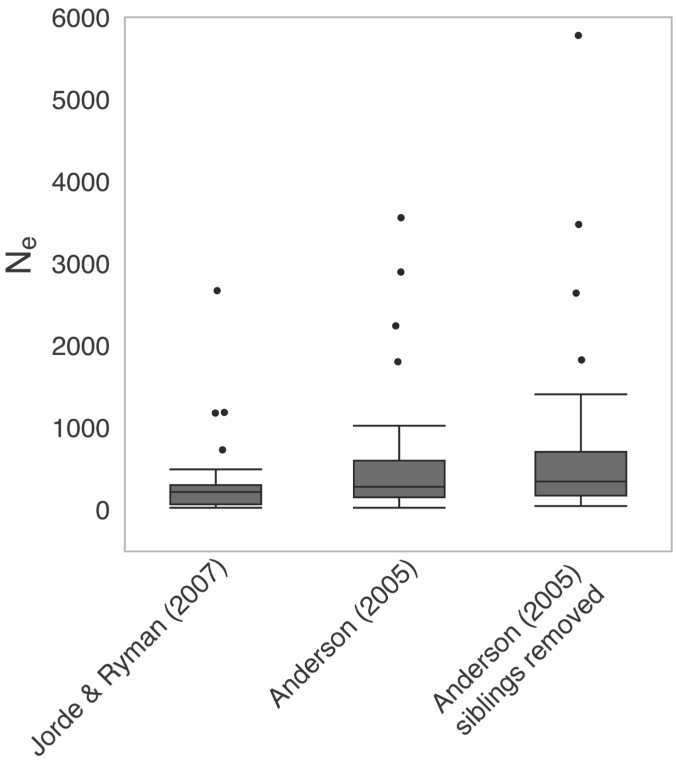

Figure S5. See also Figure 2 and S4. Box plots comparing mean N_e estimates from the Jorde and Ryman (2007) and Anderson (2005) methods as presented in Table 1, and with siblings removed. Siblings were identified using COLONY v2.0.6.3 (Jones and Wang, 2010). Box-plot boxes represent the 1st and 3rd quartile, whiskers represent the upper and lower 95 percentiles, and dots represent outliers.

Supplemental Tables

Table S1. See also Table 1. Sample information including locality, year and month of collection (year-month), generations per year estimated based on the number of months of the year wherein monthly average minimum temperature was above 10˚C in 2013 (gen/yr), number of samples included in the microsatellite data set (N), the number of siblings detected with COLONY v2.0.6.3 (Jones and Wang, 2010) in the microsatellite data set (N siblings), the number of microsatellite alleles present in the samples (N alleles), and the number of samples included in the single nucleotide polymorphism data set (N SNPs).

Locality	Year-Month	gen/year	N	N siblings	N alleles	N SNPs
01 Madera, USA	2013	6	72	0	48	
	2015		40	0	47	
02 Tucson, USA	2012	7	54	5	54	
	2013		53	0	52	
	2015		54	0	58	
03 Houston, USA [a]	2009	9	29	13	40	
	2011		19	5	37	
04 New Orleans, USA	2011	9	46	9	62	
	2012		63	1	75	11
	2014		56	0	68	
	2015		32	0	59	11
05 Vaca Keys, USA	2006	12	43	1	59	
	2009		42	6	57	
	2015		48	8	59	
06 Key West, USA	2009	12	31	1	65	12
	2011		29	7	58	
	2013		51	11	61	
	2016		52	5	61	12
07 Amacuzac, MX	2012	12	54	3	52	
	2013-Sept		54	7	48	
	2014		53	12	48	
	2016		52	8	46	
08 Coatzacoalcos, MX [a]	2003	12	35	6	44	
	2008		50	5	31	
09 Pijijiapan, MX	2006	12	48	6	33	
	2008		47	2	40	
10 Patillas, PR [b]	2012	12	54	3	57	
	2014		54	27	53	
11 Jacobina, BR	2013	12	60	0	51	37
	2014-May		60	19	50	15
	2015-Nov		59	14	52	14
12 Cachoeiro, BR [a]	2008	12	23	0	45	
	2010		47	0	52	
	2012		47	1	49	
13 Goudiry, SE [a]	2007	12	46	5	34	
	2012		54	0	53	
14 Yaounde, CM	2009-Sept	12	46	0	81	
	2014-April		54	19	82	15
	2015-June		54	17	84	16
15 Lunyo, UG [a]	2012	12	54	36	69	
	2013		53	31	73	
16 Rabai, KE	2006	12	31	2	95	
	2009		37	0	101	
	2012		16	4	50	

17 Cairns, AU	2009<br>2013-Jan<br>2015-March	12	47<br>51<br>45	14<br>1<br>5	49<br>51<br>47	

[a] Locality with evidence of temporal shifts determined by principal components analysis (Figure S1) and neighbor-joining phylogenetic analysis (Figure S2).

Table S2. See also Table 1 and 2. One-sample linkage disequilibrium based N_e estimates from *NeEstimator v2* (Do et al, 2014) showing locality, sampled time points, microsatellite (µsat) based effective population size estimates ($N_e^{\mu sat}$) with upper and lower parameteric 95% confidence intervals ($CI^{\mu sat}$), and when available, the SNP based effective population size estimates (N_e^{SNP}) with upper and lower parameteric 95% confidence intervals (CI^{SNP}).

**Locality**	**Year-Month**	**LD $N_e^{\mu sat}$**	**Lower $CI^{\mu sat}$**	**Upper $CI^{\mu sat}$**	**LD N_e^{SNP}**	**Lower CI^{SNP}**	**Upper CI^{SNP}**
01 Madera, USA	2013	69.4	44.9	124.4			
	2015	187.3	57.8	∞			
02 Tucson, USA	2012	14.6	11.5	18.6			
	2013	834.6	121.0	∞			
	2015	262.1	98.3	∞			
03 Houston, USA [a]	2009	2.0	1.6	2.6			
	2011	5.6	2.7	11.0			
04 New Orleans, USA	2011	35.0	25.7	50.6			
	2012	158.9	95.8	383.1	74.1	73.3	74.9
	2014	2526.3	166.6	∞			
	2015	32.9	21.1	61.0	13.1	13.1	13.1
05 Vaca Keys, USA	2006	82.9	47.5	222.4			
	2009	30.1	21.7	44.3			
	2015	21.4	16.6	27.9			
06 Key West, USA	2009	60.9	34.5	174.3	9.0	9.0	9.0
	2011	13.3	9.5	19.1			
	2013	25.9	20.2	33.8			
	2016	46.2	33.2	69.0	10.1	10.0	10.1
07 Amacuzac, MX	2012	27.3	20.3	38.0			
	2013-Aug	18.1	13.6	24.4			
	2014	9.9	7.3	12.9			
	2016	30.4	20.9	47.0			
08 Coatzacoalcos, MX [a]	2003	12.5	8.4	18.5			
	2008	4.1	3.0	8.9			
09 Pijijiapan, MX	2006	17.3	10.2	30.3			
	200	25.4	16.3	42.7			
10 Patillas, PR [b]	2012	59.1	39.8	99.8			
	2014	5.4	3.8	7.4			
11 Jacobina, BR	2013	359.8	105.3	∞	51.7	51.6	51.8
	2014	14.3	11.1	18.4	1.8	1.8	1.8
	2015	10.5	8.0	13.5	1.0	1.0	1.0
12 Cachoeiro, BR [a]	2008	55.2	21.7	∞			
	2010	34.5	24.0	53.4			
	2012	27.0	18.9	40.4			
13 Goudiry, SE [a]	2007	8.4	4.0	13.5			
	2012	13.7	10.7	17.4			
14 Yaounde, CM	2009-Sept	54.3	41.0	76.3			
	2014-April	19.4	16.7	22.7	3.4	3.4	3.5
	2015-June	23.4	20.0	27.5	0.9	0.9	0.9

15 Lunyo, UG [a, b]	2012	6.8	5.4	8.2			
	2013	3.4	3.1	3.7			
16 Rabai, KE	2006	32.6	25.2	44.4			
	2009	∞	279.6	∞			
	2012	1.4	1.1	1.7			
17 Cairns, AU	2009	13.0	9.7	17.2			
	2013-Jan	55.0	34.7	104.9			
	2015-March	17.9	12.8	25.5			

[a] Locality with evidence of temporal shifts determined by principal components analysis (Figure S1) and neighbor-joining phylogenetic analysis (Figure S2).

Table S3. Relates to Discussion Section. N_e estimates with the moment based temporal method of Waples (1989) presented in previous studies; locality and literature source, the time interval spanning the two samples in generations (I), microsatellite based N_e estimates (N_e^{usats}) with lower and upper 95% confidence intervals (CI^{usats}), and SNP based N_e estimates (N_e^{SNPs}) with lower and upper 95% confidence intervals (CI^{SNPs}).

**Locality** [source]	**I**	$N_e^{\mu sat}$	**Lower CI** $^{\mu sat}$	**Upper CI** $^{\mu sat}$	N_e^{SNP}	**Lower CI** ^{SNP}	**Upper CI** ^{SNP}
Chillagoe and Ingham, Australia (wet season) [a]	9	5,546	40	238			
Mossman, Mareeba…, Australia (wet season) [a]	9	298	155	575			
Gordonvale, Mossman…, Australia (dry season) [a]	9	202	98	431			
Cardwell, Tully…, Australia (dry season) [a]	9	241	104	707			
Chillagoe, Australia (dry season) [a]	9	25	11	57			
Charters Towers, Australia (dry season) [a]	9	5564	60	∞			
Yogyakrta, Indonesia (Site 6) [b]	10	367	212	563	136	128	144
Yogyakrta, Indonesia (Site 7) [b]	10	∞	∞	∞	281	264	298
Yogyakrta, Indonesia (Site 8) [b]	10	820	449	1303	730	689	773
Thailand (Village aa) [c]	9	161	60	623			
Thailand (Village a) [c]	9	93	38	234			
Thailand (Village b) [c]	9	221	66	4830			
Thailand (Village c) [c]	9	150	52	729			
Thailand (Village 6) [c]	9	206	72	1247			

[a] Summarized from Endersby et al. (2011).
[b] Summarized from Rašić et al. (2015).
[c] Summarized from Olanratmanee et al. (2013).

Table S4. See also Table 1 and Figure S5. Two-sample N_e estimates based on 12 microsatellites after sibling removal; locality, sampled years and sampled generations in parentheses counting from zero at the first time point sampled, the original harmonic mean sample size (N_O), the number of siblings removed (N_S), the final harmonic mean of sample size after sibling removal (N_F), and N_e estimates made with the Anderson (2005) method in *CoNe* (Anderson, 2005) with lower and upper 95% confidence intervals (CI).

Locality	Sampled years (generations)	N_O	N_S	N_F	N_e	Lower CI	Upper CI
01 Madera, USA	2013 & 2015 (0 & 12)	51.4	0	51.4	551.4	224.0	4850.8
02 Tucson, USA	2012 & 2013 (0 & 7)	53.5	5	50.9	97.4	60.5	168.1
	2012 & 2015 (0 & 21)	54.0	5	51.4	437.1	266.8	785.4
	2013 & 2015 (7 & 21)	53.5	0	53.5	∞	∞	∞
03 Houston, USA [a]	2009 & 2011 (0 & 18)	23.0	18	14.9	48.9	31.5	79.0
04 New Orleans, USA	2011 & 2012 (0 & 9)	53.2	10	46.3	2632.1	520.5	∞
	2011 & 2014 (0 & 27)	50.5	9	44.6	∞	∞	∞
	2011 & 2015 (0 & 36)	37.7	9	34.3	635.9	361.3	1344.3
	2012 & 2014 (9 & 27)	59.3	1	58.8	970.2	499.2	2902.5
	2012 & 2015 (9 & 36)	42.4	1	42.2	406.8	244.5	746.5
	2014 & 2015 (27 & 36)	40.7	0	40.7	197.0	108.0	460.9
05 Vaca Keys, USA	2006 & 2009 (0 & 36)	42.5	7	38.8	552.6	328.6	1037.2
	2006 & 2015 (0 & 84)	45.4	9	41.0	1819.2	1046.2	3825.2
	2009 & 2015 (36 & 84)	44.8	14	37.9	676.3	428.1	1152.5
06 Key West, USA	2009 & 2011 (0 & 24)	30.0	8	25.4	591.7	275.3	2920.7
	2009 & 2013 (0 & 48)	38.8	12	34.6	932.0	542.7	1907.2
	2009 & 2016 (0 & 84)	38.8	6	36.6	3467.2	1536.6	24485.6
	2011 & 2013 (24 & 48)	37.2	18	28.6	374.4	220.8	740.4
	2011 & 2016 (24 & 84)	37.2	12	30.0	1386.7	741.2	3610.5
	2013 & 2016 (48 & 84)	52.0	16	43.8	1163.1	610.7	3209.1
07 Amacuzac, MX	2012 & 2013 (0 & 16)	54.0	10	48.9	234.2	136.0	446.8
	2012 & 2014 (0 & 24)	53.5	15	45.5	283.4	169.1	509.0
	2012 & 2016 (0 & 48)	53.0	11	47.2	598.9	344.2	1134.9
	2013 & 2014 (16 & 24)	53.5	19	43.8	80.8	49.8	136.7
	2013 & 2016 (16 & 48)	53.0	15	45.5	320.3	192.7	552.6
	2014 & 2016 (24 & 48)	52.5	20	42.4	210.0	114.3	∞
08 Coatzacoalcos, MX [a]	2003 & 2008 (0 & 60)	41.2	11	35.3	66.0	46.9	92.3
09 Pijijiapan, MX	2006 & 2008 (0 & 24)	47.5	8	43.4	176.0	108.3	290.0
10 Patillas, PR	2012 & 2014 (0 & 24)	54.0	30	35.3	226.5	137.8	400.1
11 Jacobina, BR	2013 & 2014 (0 & 8)	60.5	19	49.4	105.8	66.1	178.6
	2013 & 2015 (0 & 14)	59.5	14	51.4	269.8	163.6	505.6
	2014 & 2015 (8 & 14)	60.0	33	43.4	66.1	43.3	104.8
12 Cachoeiro, BR [a]	2008 & 2010 (0 & 24)	30.9	0	30.9	175.2	118.8	267.8
	2008 & 2012 (0 & 48)	30.9	1	30.7	712.3	417.7	1441.2
	2010 & 2012 (24 & 48)	47.0	1	46.5	106.2	76.4	149.0
13 Goudiry, SE [a]	2007 & 2012 (0 & 60)	49.7	5	46.6	150.7	117.3	192.8
14 Yaounde, CM	2009 & 2014 (0 & 55)	50.3	19	40.1	646.0	468.1	923.1
	2009 & 2015 (0 & 69)	50.7	17	42.0	1403.3	941.1	2267.9
	2014 & 2015 (55 & 69)	54.5	36	36.4	314.8	201.7	559.9
15 Lunyo, UG [a]	2012 & 2013 (0 & 12)	53.5	67	19.8	83.2	57.7	126.5
16 Rabai, KE	2006 & 2009 (0 & 36)	33.7	2	32.5	5767.1	1583.2	∞
	2006 & 2012 (0 & 72)	21.1	6	17.0	270.6	182.0	420.4
	2009 & 2012 (36 & 72)	22.3	4	18.1	145.4	96.7	229.6
17 Cairns, AU	2009 & 2013 (0 & 48)	49.5	15	40.5	716.8	438.9	1285.3
	2009 & 2015 (0 & 62)	46.5	19	36.8	625.1	387.3	1080.3
	2009 & 2015 (0 & 62)	47.8	6	44.4	187.4	108.8	364.7

[a] Locality with evidence of temporal shifts determined by principal components analysis (Figure S1) and neighbor-joining phylogenetic analysis (Figure S2).
